# Inaugural Review Prize 2023: The exercise hyperpnoea dilemma: A 21st‐century perspective

**DOI:** 10.1113/EP091506

**Published:** 2024-03-29

**Authors:** Joseph F. Welch, Gordon S. Mitchell

**Affiliations:** ^1^ School of Sport, Exercise and Rehabilitation Sciences, College of Life and Environmental Sciences University of Birmingham Edgbaston Birmingham UK; ^2^ Breathing Research and Therapeutics Centre, Department of Physical Therapy, McKnight Brain Institute University of Florida Gainesville Florida USA

**Keywords:** cerebellum, chemogenetics, exercise, hyperpnoea, learning, optogenetics, plasticity

## Abstract

During mild or moderate exercise, alveolar ventilation increases in direct proportion to metabolic rate, regulating arterial CO_2_ pressure near resting levels. Mechanisms giving rise to the hyperpnoea of exercise are unsettled despite over a century of investigation. In the past three decades, neuroscience has advanced tremendously, raising optimism that the ‘exercise hyperpnoea dilemma’ can finally be solved. In this review, new perspectives are offered in the hope of stimulating original ideas based on modern neuroscience methods and current understanding. We first describe the ventilatory control system and the challenge exercise places upon blood‐gas regulation. We highlight relevant system properties, including feedforward, feedback and adaptive (i.e., plasticity) control of breathing. We then elaborate a seldom explored hypothesis that the exercise ventilatory response continuously adapts (learns and relearns) throughout life and ponder if the memory ‘engram’ encoding the feedforward exercise ventilatory stimulus could reside within the cerebellum. Our hypotheses are based on accumulating evidence supporting the cerebellum's role in motor learning and the numerous direct and indirect projections from deep cerebellar nuclei to brainstem respiratory neurons. We propose that cerebellar learning may be obligatory for the accurate and adjustable exercise hyperpnoea capable of tracking changes in life conditions/experiences, and that learning arises from specific cerebellar microcircuits that can be interrogated using powerful techniques such as optogenetics and chemogenetics. Although this review is speculative, we consider it essential to reframe our perspective if we are to solve the till‐now intractable exercise hyperpnoea dilemma.

## Abstract (highlights)

- **What is the topic of this review?**
  This review reframes our perspective on the control of breathing during mild or moderate exercise, emphasising concepts of learning, memory and plasticity.
- **What advances does it highlight?**
  We propose that exercise hyperpnoea continuously adapts to ever‐changing life conditions/experiences. We question if the memory ‘engram’ encoding exercise hyperpnoea resides within the cerebellum and outline powerful new methods to test this hypothesis. It is our hope that this review generates new ideas and renewed study of exercise hyperpnoea in the 21st century.

## 1 INTRODUCTION

Exercise is the greatest ventilatory stimulus routinely encountered in life. The hyperpnoea of exercise is a vital homeostatic response that must be robust and adaptable. Despite the physiological significance of exercise hyperpnoea, neural mechanisms underpinning the near‐precise matching of alveolar ventilation to metabolic rate (i.e., Δ${\dot{V}}_{A}$ ∝ Δ${\dot{V}}_{CO_{2}}$) with only minor alterations in arterial blood‐gas tensions (Douglas & Haldane, 1909; Douglas et al., 1912; Haldane & Priestley, 1905) remain enigmatic. Consequently, exercise hyperpnoea has been termed the ‘unanswered question’ (Whipp, 2008), the ‘ultra‐secret’ (Grodins, 1981) and the ‘holy grail’ (Dempsey, 2006) of respiratory and exercise physiology.

By the end of the 19th century, German physiologists Julius Geppert and Nathan Zuntz articulated possible neural circuits and sources of stimuli involved in the control of breathing during exercise (Geppert & Zuntz, 1888; Zuntz & Geppert, 1886). In the 20th century, Dejours (1964), Grodins (1950) and Wasserman et al. (1979) provided updated theories at decade intervals on the nature of exercise hyperpnoea. Postulated factors were initially supported and then denied, generating more questions than answers. Since the late 1980s, major advances in our understanding of exercise hyperpnoea have been limited, despite continuing (but waning) interest (Bruce, 2022; Bruce et al., 2019; Casaburi, 2012; Dempsey et al., 2022; Forster et al., 2012; Haouzi, 2020; Parkes, 2017; Ward, 2019).

Over the past three decades, tremendous technological and conceptual advances in neuroscience have been made, including increased awareness of the capacity for neuroplasticity in respiratory control (Fuller & Mitchell, 2017; Mitchell & Babb, 2006; Mitchell & Baker, 2022; Vose et al., 2022). With increased knowledge, new ideas concerning exercise hyperpnoea have emerged, accompanied by powerful new techniques to test those ideas. The purposes of this review are to (a) define what is known about exercise hyperpnoea, its underlying mechanisms and the neural system through which it operates, and (b) pose a novel hypothesis that cerebellar learning is obligatory to develop a ‘normal’ exercise ventilatory response that continuously adapts (learns and relearns) to meet the demands on pulmonary ventilation and gas exchange imposed by the ever‐changing physiological conditions experienced throughout life.

## 2 PART I: THE VENTILATORY CONTROL SYSTEM

**FIGURE 1:**
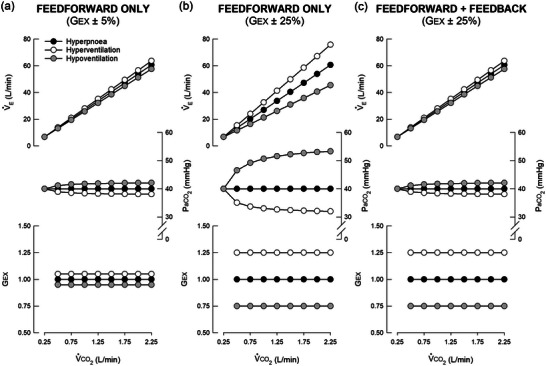
Feedforward and feedback interactions to exercise hyperpnoea. (a) Illustration of very good regulation of arterial CO_2_ pressure ($P_{\mathrm{aC}O_{2}}$) with small (5%) deviations in the feedforward exercise gain (*G*_EX_) from an ‘ideal gain’ that would generate isocapnia (black symbols); there are corresponding 5% deviations in the system gain (${\dot{V}}_{E}$:${\dot{V}}_{CO_{2}}$). Slight increases in slope produce hypocapnia (white symbols), whereas slight decreases in slope produce modest hypercapnia (grey symbols). In this case, a 5% increase or decrease in *G*_EX_ above or below the isocapnic value would alter $P_{\mathrm{aC}O_{2}}$ by 2 mmHg from its resting level (40 mmHg) at a ${\dot{V}}_{CO_{2}}$ of 2.25 L/min (10‐fold above rest). (b) The hypothetical $P_{\mathrm{aC}O_{2}}$ regulation expected with larger (25%) deviations in *G*_EX_ from ‘ideal’, with corresponding deviations in the ${\dot{V}}_{E}$:${\dot{V}}_{CO_{2}}$ relationship; in this simulation, an absence of chemofeedback is assumed. Such 25% variances in *G*_EX_ would decrease $P_{\mathrm{aC}O_{2}}$ by 8 mmHg (with excess *G*_EX_) or increase $P_{\mathrm{aC}O_{2}}$ by 13 mmHg (with inadequate *G*_EX_) from resting levels. (c) Restoration of very good (not perfect) $P_{\mathrm{aC}O_{2}}$ regulation with 25% deviations in *G*_EX_ but with CO_2_ chemofeedback layered onto the feedforward drive, thereby minimising errors in $P_{\mathrm{aC}O_{2}}$ regulation. With the combination, only a small shift (5%) in the ${\dot{V}}_{E}$:${\dot{V}}_{CO_{2}}$ slope from an isocapnic value occurs despite 25% excessive or inadequate feedforward drive. Thus, as in (a), $P_{\mathrm{aC}O_{2}}$ is once again regulated within ∼2 mmHg of its resting value—a response difficult to discriminate from isocapnia without repeated $P_{\mathrm{aC}O_{2}}$ measurements. For all calculations in this simulation, the dead space to tidal volume ratio was kept at 0.2. ${\dot{V}}_{CO_{2}}$, rate of CO_2_ production; ${\dot{V}}_{E}$, minute ventilation.

The ventilatory response to mild or moderate steady‐state exercise consists of three distinct phases. Phase I describes an immediate (within one pedal stroke or step) increase in minute ventilation (${\dot{V}}_{E}$; see Table 1 for list of frequently used abbreviations) at exercise onset. Phase II describes an exponential increase in ${\dot{V}}_{E}$ with a time constant of 60–90 s. Phase III describes a ${\dot{V}}_{E}$ plateau (i.e., steady‐state) beginning approximately 3 min after exercise onset. A successful exercise hyperpnoea is defined by regulation of arterial $P_{CO_{2}}$ ($P_{\mathrm{aC}O_{2}}$) near its set‐point (∼40 mmHg) due to proportional increases in ${\dot{V}}_{A}$ and ${\dot{V}}_{CO_{2}}$ (Dempsey & Welch, 2023). However, it must be appreciated that good $P_{\mathrm{aC}O_{2}}$ regulation (i.e., ±2 mmHg of rest) is not evidence of a single mechanism that gives rise to precise ${\dot{V}}_{A}$:${\dot{V}}_{CO_{2}}$ coupling. For example, even a 25% dissociation between the primary drive to ${\dot{V}}_{A}$ and ${\dot{V}}_{CO_{2}}$ yields only an ∼2 mmHg change in $P_{\mathrm{aC}O_{2}}$ (Bennett & Fordyce, 1985) due to chemofeedback that ‘layers’ onto the primary (feedforward) drive to breathe once $P_{\mathrm{aC}O_{2}}$ regulation is lost (see Figure 1). Indeed, it is very difficult to distinguish between precise ${\dot{V}}_{A}$:${\dot{V}}_{CO_{2}}$ feedforward coupling versus a moderate mismatch refined by chemofeedback. In most vertebrate species, mild hypocapnia is observed during mild or moderate steady‐state exercise, whereas humans exhibit slight hypercapnia (Clark et al., 1980; Dempsey et al., 1984; Fordyce et al., 1982). Hence, despite widespread use of the term ‘isocapnic hyperpnoea’ to characterise a human exercise ventilatory response, we suggest that the terms ‘isocapnia’ and ‘hyperpnoea’ should be used with caution since the precision of $P_{\mathrm{aC}O_{2}}$ regulation varies across species and with exercise intensity.

**TABLE 1:** List of abbreviations.

Abbreviation	
*G*_EX_	Exercise gain
*G*_SYS_	System gain
LTM	Long‐term modulation
$P_{\mathrm{aC}O_{2}}$	Arterial CO_2_ partial pressure
$P_{aO_{2}}$	Arterial O_2_ partial pressure
pH_a_	Arterial pH
*S*	Feedback gain
STM	Short‐term modulation
${\dot{V}}_{A}$	Alveolar ventilation
${\dot{V}}_{CO_{2}}$	Rate of CO_2_ production
${\dot{V}}_{O_{2}}$	Rate of O_2_ consumption
*V*_D_	Dead space volume
${\dot{V}}_{E}$	Minute ventilation
*V*_T_	Tidal volume

### 2.1 Operational model of the ventilatory control system

**FIGURE 2:**
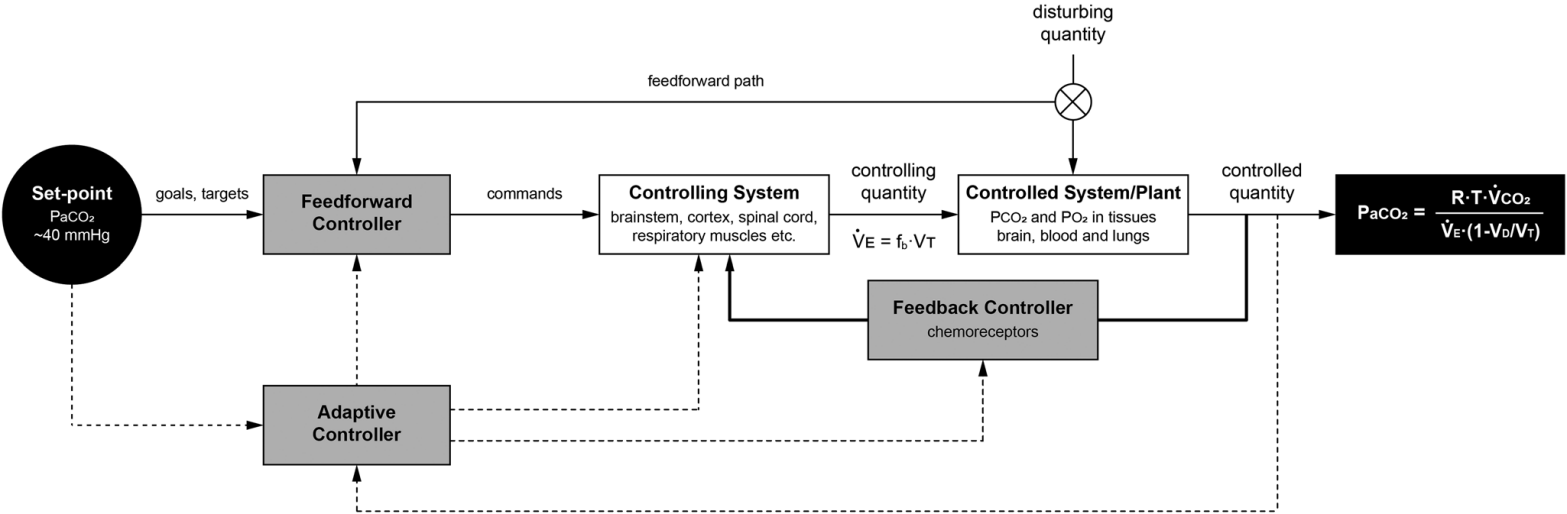
Operational model of the ventilatory control system. See text for description. Dashed lines indicate adaptive controller pathways. Thick black lines indicate feedback loop. *f*_b_, breathing frequency; $P_{\mathrm{aC}O_{2}}$, arterial CO_2_ partial pressure; $P_{aO_{2}}$,  arterial O_2_ partial pressure; $P_{CO_{2}}$, CO_2_ partial pressure; $P_{O_{2}}$, O_2_ partial pressure; ${\dot{V}}_{CO_{2}}$, rate of CO_2_ production; *V*_D_, dead space volume; ${\dot{V}}_{E}$, minute ventilation; *V*_T_, tidal volume.

An operational model of the ventilatory control system is illustrated in Figure 2; this simplified model is based upon control theory as described by Houk (1988) and mathematical simulations of the exercise stimulus described by Fred Grodins (Grodins et al., 1954; Grodins et al., 1967). Understanding mechanisms controlling physiological systems requires knowledge of the regulated variables. During mild or moderate exercise, the primary regulated variable is $P_{\mathrm{aC}O_{2}}$. The relationships between the controlled ($P_{\mathrm{aC}O_{2}}$), controlling (${\dot{V}}_{A}$) and disturbing variables (${\dot{V}}_{CO_{2}}$) are summarised in the alveolar ventilation equation:

(1) $$P_{\mathrm{aC}O_{2}}=\;\frac{R\times T\times {\dot{V}}_{CO_{2}}}{{\dot{V}}_{A}}$$

where *R* is the gas constant (2.785 [mL BTPS mmHg]/[mL STPD K]) and *T* is temperature in K. This assumes only conservation of mass, steady‐state conditions and an inspired CO_2_ fraction of zero.

Since the alveolar volume is equal to the tidal volume minus the physiological dead space (i.e., ${\dot{V}}_{A}$ = *V*_T_ – *V*_D_), ${\dot{V}}_{A}$ can be replaced with ${\dot{V}}_{E}$:

(2) $$P_{\mathrm{aC}O_{2}}=\;\frac{R\times T\times {\dot{V}}_{CO_{2}}}{{\dot{V}}_{E}\left(1-V_{D}/V_{T}\right)}$$

Figure 1 illustrates that isocapnia from rest to exercise can be achieved if ${\dot{V}}_{E}$ and ${\dot{V}}_{CO_{2}}$ increase in direct proportion, with a slope equal to *RT*/[$P_{\mathrm{aC}O_{2}}$(1 – *V*_D_/*V*_T_)] (Mitchell, 1990; Mitchell et al., 1984). Deviations in the ${\dot{V}}_{E}$:${\dot{V}}_{CO_{2}}$ slope from this value, even if linear, lead to hypocapnia or hypercapnia. Equation 2 is extremely important in understanding exercise hyperpnoea since it expresses all possible system solutions during steady‐state (phase III) exercise.

#### 2.1.1 Controlling system

Typically, $P_{\mathrm{aC}O_{2}}$ is regulated near a set‐point of ∼40 mmHg. With an increase in ${\dot{V}}_{CO_{2}}$, $P_{\mathrm{aC}O_{2}}$ can be defended only by changes in neural respiratory motor output that drive appropriate increases in ${\dot{V}}_{E}$. The neural control system (i.e., central controller) includes supra‐pontine (e.g., primary motor cortex, periaqueductal grey, etc.) and ponto‐medullary (e.g., ventral respiratory column) circuits that subserve voluntary (i.e., behavioural) and automatic breathing, respectively. These neural circuits activate more than 60 different respiratory muscles to produce changes in airflow with an inherent frequency and amplitude. The total volume of air displaced per minute in response to forcing functions generated by the central controlling system is the controlling quantity, or ${\dot{V}}_{E}$, given by the product of *V*_T_ and breathing frequency. Minute ventilation is determined by coordinated activation of spinal respiratory pump muscles (e.g., diaphragm, intercostal and abdominal muscles) and cranial upper airway resistance muscles (e.g., genioglossus) (Pilarski et al., 2019). The timing and pattern of respiratory muscle recruitment during exercise follows the ‘principle of minimum effort’ (Otis et al., 1950) to minimise respiratory muscle work.

The primary function of the ventilatory control system during exercise is to regulate $P_{\mathrm{aC}O_{2}}$ at or near its resting level. In this context, the term ‘feedback’ is defined with respect to errors in $P_{\mathrm{aC}O_{2}}$ regulation. Thus, chemofeedback mechanisms sense changes in $P_{\mathrm{aC}O_{2}}$ and/or arterial pH, driving or inhibiting ${\dot{V}}_{E}$ to curtail $P_{\mathrm{aC}O_{2}}$ deviations from its set‐point. For the purposes of this review, feedback does not refer to known sensory inputs from the lungs (e.g., pulmonary stretch receptors) or exercising limbs (e.g., metaboreceptors). If $P_{\mathrm{aC}O_{2}}$ deviates from rest due to excessive or inadequate feedforward drives to breathe, chemofeedback minimises the error in $P_{\mathrm{aC}O_{2}}$ regulation.

In contrast, ‘feedforward’ stimuli refers to neural drives to breathe that predict or anticipate the need for increased ${\dot{V}}_{E}$ in response to increased ${\dot{V}}_{CO_{2}}$, thereby regulating $P_{\mathrm{aC}O_{2}}$ without need for chemofeedback. In other words, feedforward commands translate goals, targets and information about potential disturbances to $P_{\mathrm{aC}O_{2}}$ that have not yet occurred (Houk, 1988). The distinction between feedforward versus feedback is an essential premise of this review and is defined by whether a neural mechanism is activated directly by changes in $P_{\mathrm{aC}O_{2}}$. For example, $P_{\mathrm{aC}O_{2}}$ changes are not known to alter sensory afferent inputs from the lung or muscle, but do directly alter chemoreceptor inputs. Whilst feedforward and feedback control offer short‐term protection that minimises errors in $P_{\mathrm{aC}O_{2}}$ regulation, adaptive control provides a longer‐lasting solution to accommodate changing conditions, as discussed later in this review.

#### 2.1.2 Controlled system

The part of the system that is controlled is referred to as the ‘plant’. The ventilatory control plant includes structures and processes involved in pulmonary gas exchange (e.g., alveoli, pulmonary capillaries) that directly influence $P_{O_{2}}$ and $P_{CO_{2}}$ in the lungs, tissues, blood and brain. The plant is disrupted by factors such as physiological dead space (expressed as *V*_D_/*V*_T_), pulmonary mechanics (compliance or resistance), inspired $P_{O_{2}}$ and $P_{CO_{2}}$, and the respiratory exchange ratio (${\dot{V}}_{CO_{2}}$/${\dot{V}}_{O_{2}}$).

During mild or moderate exercise, $P_{\mathrm{aC}O_{2}}$ is tightly regulated and, as a result, arterial $P_{O_{2}}$ ($P_{aO_{2}}$) and pH (pH_a_) remain close to resting values. The relationship between alveolar $P_{CO_{2}}$ and $P_{O_{2}}$ is given by the alveolar gas equation (Fenn et al., 1946):

(3) $$P_{AO_{2}}=P_{IO_{2}}-\frac{P_{\mathrm{AC}O_{2}}}{\mathrm{RQ}}$$

where $P_{AO_{2}}$ is alveolar $P_{O_{2}}$, $P_{IO_{2}}$ is inspired $P_{O_{2}}$, $P_{\mathrm{AC}O_{2}}$ is alveolar $P_{CO_{2}}$ and RQ is respiratory quotient, ${\dot{V}}_{CO_{2}}$/${\dot{V}}_{O_{2}}$ .

In Equation 3, alveolar and arterial $P_{CO_{2}}$ are reasonable approximations of one another; however, a similar assumption cannot be made for alveolar and arterial $P_{O_{2}}$. The relationship between $P_{\mathrm{aC}O_{2}}$ and pH_a_ is formally expressed by the Henderson–Hasselbalch equation applied to the hydration/dehydration equilibrium of CO_2_:

(4) $$pH_{a}=pK+\log\left[\frac{\mathrm{HC}O_{3^{-}}}{0.03\times P_{\mathrm{aC}O_{2}}}\right]$$

where pK is the collective dissociation constant (6.1) and 0.03 is the solubility for CO_2_ in plasma.

Thus, since alveolar (and arterial) $P_{CO_{2}}$ is inversely related to alveolar $P_{O_{2}}$ and pH_a_, careful $P_{\mathrm{aC}O_{2}}$ regulation achieves satisfactory regulation of alveolar $P_{O_{2}}$ and pH_a_.

## 3 PART II: LAYERS OF REGULATION

**FIGURE 3:**
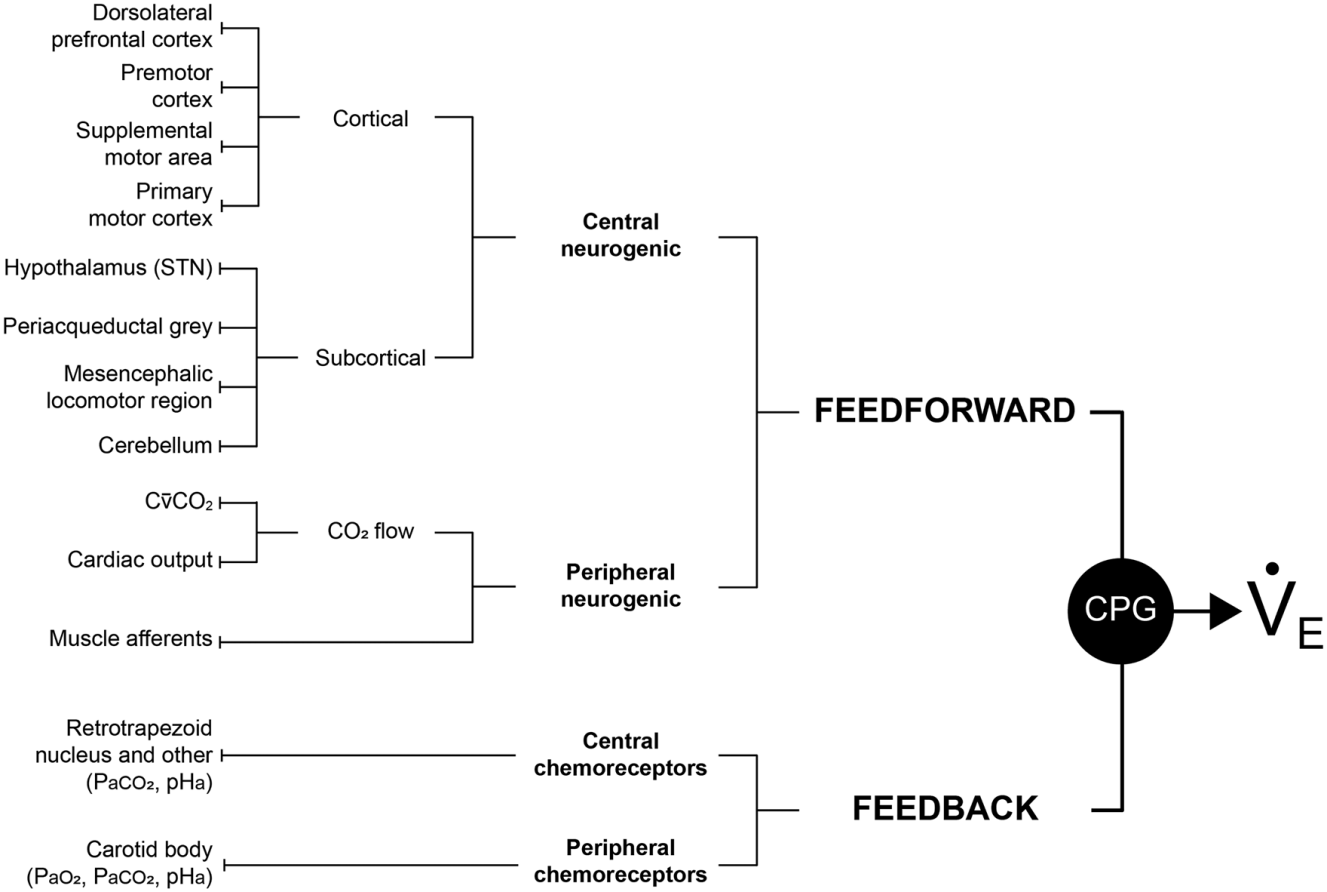
Sources of feedforward and feedback stimuli. The exercise ventilatory response results from combined (layered) feedforward and feedback drives to the brainstem respiratory central pattern generator (CPG). Many sources of feedforward and sensory stimuli have been identified, and are listed on the left. Muscle afferents include sensory afferent neurons from both respiratory and locomotor muscles. CPG, respiratory central pattern generators; $C_{\bar{v}CO_{2}}$, mixed‐venous CO_2_ content; $P_{\mathrm{aC}O_{2}}$, arterial CO_2_ partial pressure; $P_{aO_{2}}$, arterial O_2_ partial pressure; pH_a_, arterial pH; STN, subthalamic nucleus; ${\dot{V}}_{E}$, minute ventilation. Adapted from Cunningham et al. (1986).

Based on the operational model of the ventilatory control system presented in Figure 2, three major control strategies (defined in Table 2), representing independent but interacting layers of regulation, govern the integrated exercise ventilatory response. Strategies include (a) feedback, (b) feedforward, and (c) adaptive control. Figure 3 illustrates some major identified sources of feedforward and feedback contributions to exercise hyperpnoea. Evidence for each control strategy in $P_{\mathrm{aC}O_{2}}$ regulation during exercise is described below; interested readers are directed elsewhere for more comprehensive discussion (Forster et al., 2012).

**TABLE 2:** List of definitions.

Term	Definition
Adaptive control	$P_{\mathrm{aC}O_{2}}$ regulation due to short‐ and long‐term adjustments in the moment‐to‐moment properties of the ventilatory control system
Central neurogenic	A feedforward stimulus for $P_{\mathrm{aC}O_{2}}$ regulation originating from cortical and subcortical structures
Exercise hyperpnoea	$P_{\mathrm{aC}O_{2}}$ regulation about its set‐point due to proportional changes in ${\dot{V}}_{A}$ and ${\dot{V}}_{CO_{2}}$
Feedback control	Closed‐loop chemosensory regulation of $P_{\mathrm{aC}O_{2}}$
Feedforward control	Open‐loop $P_{\mathrm{aC}O_{2}}$ regulation by predictive or corrective commands
Long‐term modulation	A persistent change in the exercise ventilatory response that outlasts the inducing stimulus
Peripheral neurogenic	A feedforward stimulus for $P_{\mathrm{aC}O_{2}}$ regulation originating from peripheral sensory receptors
Short‐term modulation	An immediate change in the exercise ventilatory response that is reversed upon removal of the inducing stimulus

Abbreviations: ${\dot{V}}_{A}$, alveolar ventilation; ${\dot{V}}_{CO_{2}}$, rate of CO_2_ production.;$P_{\mathrm{aC}O_{2}}$, arterial CO_2_ partial pressure.

### 3.1 Feedback control

Feedback control operates as closed‐loop chemosensory regulation of $P_{\mathrm{aC}O_{2}}$. During mild or moderate exercise in healthy humans, $P_{\mathrm{aC}O_{2}}$ is maintained close to the set‐point, defined here as the mean resting value. Thus, classical arterial chemoreception via central and peripheral chemoreceptors has been dismissed as a major contributing factor to the control of breathing during exercise at sea level (Grodins, 1950; Shea et al., 1993; Wasserman, Whipp, Koyal et al., 1975). However, in all other mammalian species (as well as other terrestrial vertebrates) studied to date, breathing increases in excess of ${\dot{V}}_{CO_{2}}$, decreasing $P_{\mathrm{aC}O_{2}}$ slightly from rest to exercise in steady‐state (Bainton, 1978; Dempsey et al., 1984; Smith et al., 1983). Thus, CO_2_ chemoreception in species other than humans actually inhibits breathing in exercise (Mitchell, 1990).

Multiple studies confirm that carotid body chemoreceptors are not necessary for a successful exercise hyperpnoea, as shown by the essentially normal exercise ventilatory responses in carotid denervated ponies (Pan et al., 1983), goats (Mitchell et al., 1984) and dogs (Bainton, 1978). However, three caveats must be acknowledged. First, carotid denervation alone increases the resting $P_{\mathrm{aC}O_{2}}$ set‐point and slightly reduces the ${\dot{V}}_{E}$:${\dot{V}}_{CO_{2}}$ slope as required in accordance with Equation 2 (Bainton, 1978; Mitchell et al., 1984). Second, carotid denervated ponies exhibit exaggerated hyperventilation during phases I and II of the exercise ventilatory response (Forster et al., 1983) with a similar but opposite (exaggerated hypoventilation) response in asthmatic humans with carotid resection (Honda et al., 1979). Third, peripheral chemoreceptors contribute tonic respiratory drive at rest and during exercise, as indicated by the ∼10% inhibition of breathing in response to a few breaths of hyperoxia (Dejours, 1962). Whilst peripheral chemoreceptors may ‘fine‐tune’ ${\dot{V}}_{E}$ by minimising blood‐gas disturbances during steady‐state transitions (Pan et al., 1983), the primary exercise stimulus is feedforward with respect to $P_{\mathrm{aC}O_{2}}$ regulation in humans and other vertebrate species.

Beyond mild to moderate exercise, peripheral chemoreceptors are a logical contributor to the hyperventilation of heavy exercise, sensing increases in core body temperature and circulating metabolites such as lactic acid, noradrenaline, adenosine, glucose and potassium (Paterson, 1992; Ward, 1994). Asthmatic patients with carotid resection (Wasserman, Whipp, Koyal et al., 1975) and children with central congenital hypoventilation syndrome (Shea et al., 1993) display normal steady‐state hyperpnoea in moderate exercise, but blunted hyperventilation in heavy exercise, signifying peripheral chemoreceptor contributions. Conversely, other reports show that the hyperventilation of heavy exercise is independent of peripheral chemoreceptors or metabolic acidosis (Pan et al., 1986). The view that changes in arterial potassium is sufficient to drive breathing during heavy exercise (Paterson, 1992; Paterson et al., 1989) is not borne out by studies in goats, since ventilatory responses to hyperkalaemia are insufficient to account for exercise hyperpnoea (Warner & Mitchell, 1990). Further, the ventilatory response to increased circulating noradrenaline is insufficient to account for exercise hyperpnoea or hyperventilation in moderate or heavy exercise (Pizarro et al., 1992; Warner & Mitchell, 1991).

### 3.2 Feedforward control

Feedforward control is open‐loop $P_{\mathrm{aC}O_{2}}$ regulation by predictive or corrective commands. The feedforward controller specifies goals or targets about the overall control process and receives sensory input from receptors that detect potential disturbances to $P_{\mathrm{aC}O_{2}}$. Literature supports at least two types of feedforward mechanism in exercise hyperpnoea: (a) central neurogenic and (b) peripheral neurogenic.

#### 3.2.1 Central neurogenic mechanisms

Central command has been defined as a feedforward mechanism involving ‘parallel activation of motor and cardiovascular centres’ (Goodwin et al., 1972). In this context, breathing should be regarded as a ‘motor’ (vs. autonomic) system. The phase I ventilatory response is remarkably fast and ‘cannot be brought about by any chemical regulation as a result of the processes in the working muscles’ (Krogh & Lindhard, 1913). Anticipation of exercise is sufficient to increase breathing in the transition from rest to work and was suggested to represent ‘irradiation of impulses from the motor cortex’ (Krogh & Lindhard, 1913). We prefer to use the term ‘central neurogenic’ (vs. central command), which we define as a feedforward stimulus with regard to $P_{\mathrm{aC}O_{2}}$ regulation that originates from cortical and/or subcortical structures of the central nervous system.

Studies using positron emission tomography to measure changes in regional cerebral blood flow as a reflection of regional neuronal activity during and/or after voluntary or imagined exercise demonstrate increased cortical and subcortical circuit activation, including the dorsolateral prefrontal cortex, premotor cortex, supplementary motor area, primary motor cortex and cerebellum (Fink et al., 1995; Thornton et al., 2001; Williamson et al., 2002; Williamson et al., 2006). Due to associations with learning and memory (formation/storage), some have postulated that activation of the aforementioned circuits may signify a learned or behavioural response at an unknown site (Fink et al., 1995; Guz, 1997; Thornton et al., 2001).

Evidence for subcortical central neurogenic contributions to breathing in exercise stem from ‘fictive’ respiration and locomotion studies in neuromuscularly blocked decorticate cats. In these studies, stimulation of the hypothalamus and mesencephalic locomotor region elicited parallel increases in phrenic and limb motor output, as well as arterial blood pressure, accompanied by a decrease in $P_{\mathrm{aC}O_{2}}$ (Eldridge et al., 1981; Eldridge et al., 1985). Another potential site of interest is the midbrain periaqueductal grey, regarded as a cognitive integrator for central and peripheral neurogenic stimuli (Mantyh, 1983; Paterson, 2014), with functional connectivity to and from higher brain regions (e.g., dorsolateral prefrontal cortex) (An et al., 1998), basal ganglia (Horiuchi et al., 2009), brainstem cardiorespiratory central pattern generators (Bandler & Carrive, 1988) and group III/IV afferents (Kramer et al., 1996). An increase in periaqueductal grey activity (i.e., local field potentials) in humans undergoing deep brain stimulation for neuropathic pain occurs in anticipation of mild exercise, mirrored by changes in heart rate and blood pressure (Green & Paterson, 2008, 2020; Green et al., 2007).

Despite extensive literature supporting cortical and subcortical sites as primary mediators of the exercise ventilatory response, evidence from studies of ${\dot{V}}_{E}$:${\dot{V}}_{CO_{2}}$ tracking during sinusoidal exercise indicates that the exercise ventilatory response can be uncoupled from limb movement (and presumably central neurogenic stimuli) (Casaburi et al., 1977; Haouzi et al., 2004). As the duration between sinusoidal oscillations in exercise workload decreases, ${\dot{V}}_{E}$ and ${\dot{V}}_{CO_{2}}$ changes also decrease but with an increased phase lag. Collectively, these observations led to the belief that the exercise ventilatory response is more closely associated with ${\dot{V}}_{CO_{2}}$ changes vs. central neural (motor) command. In agreement, the exercise ventilatory response tracks ${\dot{V}}_{CO_{2}}$ vs. limb movement in ponies (Forster et al., 1984). Whilst the relationship between ${\dot{V}}_{E}$ and ${\dot{V}}_{CO_{2}}$ (and the precision of $P_{\mathrm{aC}O_{2}}$ regulation) during treadmill exercise is similar when workload is increased via treadmill grade vs. speed, the pattern of breathing shifts in both dogs (Bainton, 1972) and goats (Smith et al., 1983). Thus, the pattern of locomotion (stride frequency vs. strength of contraction) impacts the pattern of breathing (frequency vs. volume) during exercise. Whether this shift arises from central neurogenic versus peripheral neurogenic stimuli from exercising limbs is unknown.

#### 3.2.2 Peripheral neurogenic mechanisms

Peripheral neurogenic mechanisms are a feedforward stimulus (with respect to $P_{\mathrm{aC}O_{2}}$ regulation) originating from peripheral sensory neurons. Two main peripheral neurogenic stimuli considered in the context of exercise hyperpnoea are (a) CO_2_ flux to the lung, and (b) group III/IV afferents.

By infusing CO_2_ into the femoral vein of exercising rats, Yamamoto and Edwards Jr (1960) reported a proportional increase in ${\dot{V}}_{A}$ without appreciable change in $P_{\mathrm{aC}O_{2}}$, claiming that ‘CO_2_ is sufficient stimulus for the regulation of its own concentration’. However, the authors were unable to prevent (and account for) large changes in cardiac output and blood volume during CO_2_ loading, which may independently stimulate breathing (Ponte & Purves, 1978). In follow‐up studies, some supported the findings of Yamamoto and Edwards Jr (Green & Schmidt, 1984; Wasserman et al., 1975), whereas others contradicted them (Bennett et al., 1984; Reischl et al., 1979). Carbon dioxide flow to the lungs (${\dot{Q}}_{CO_{2}}$) is given by the product of cardiac output ($\dot{Q}$) and mixed‐venous CO_2_ content ($C_{\bar{v}CO_{2}}$):

(5) $${\dot{Q}}_{CO_{2}}=\dot{Q}\times C_{\bar{v}CO_{2}}$$

For decades, it was hypothesised that an unknown mixed‐venous chemoreceptor capable of detecting changes in mixed‐venous $P_{CO_{2}}$ and/or CO_2_ flow to the lungs could account for an isocapnic exercise hyperpnoea. However, although intrapulmonary CO_2_ chemoreceptors exist in birds and reptiles (Milsom et al., 2004; Powell et al., 1988; Tallman & Grodins, 1982), analogous receptors have never been found in mammals despite considerable effort (Bartoli et al., 1973; Mitchell et al., 1980). Some authors claimed to provide evidence of venous $P_{CO_{2}}$ sensors located in the pulmonary artery (Duke et al., 1963), pulmonary C‐fibre endings (Trenchard et al., 1984), aorticopulmonary glomus tissue (Hughes, 1965) and CO_2_‐modulated pulmonary stretch receptors (Green et al., 1986), while others refuted such claims (Coleridge et al., 1966; Cropp & Comroe, 1961; Dejours et al., 1955; Fordyce & Grodins, 1980; Sylvester et al., 1973). Abrupt release of venous tourniquets elicits hyperpnoea within 4 s, but this response has been attributed to activation of cardiopulmonary baroreceptors sensitive to changes in blood volume, stretch and pressure versus changes in venous $P_{CO_{2}}$ or CO_2_ flow (Mills, 1944).

In the 1980s, Phillipson and co‐workers provided evidence supporting CO_2_ flow per se as a unique stimulus to breathing. By removing CO_2_ from the venous circulation at a rate equal to its production in resting sheep, prolonged apnoea was observed, despite maintenance of normal $P_{\mathrm{aC}O_{2}}$, pH_a_ and $P_{aO_{2}}$ (Phillipson et al., 1981b). Thus, the authors claimed breathing depends upon CO_2_ flow to the lungs by an unknown mechanism. Using extracorporeal perfusion, Phillipson et al. (1981a) infused or removed CO_2_ from the venous circulation in exercising sheep. Blood drained from the inferior vena cava and jugular veins was pumped through extracorporeal membrane lungs; ${\dot{V}}_{CO_{2}}$ was raised using 100% CO_2_ or lowered using O_2_/N_2_ mixtures, and the blood then returned to the superior vena cava. In 51 steady‐state measurements, loading or scrubbing CO_2_ with or without exercise led to proportional changes in ${\dot{V}}_{A}$ without significant change in $P_{\mathrm{aC}O_{2}}$. However, the statistical grounds on which these conclusions were made have been challenged (Bennett et al., 1984) on the basis that: (a) the use of pooled data makes it difficult to discern true isocapnia versus predicted changes in $P_{\mathrm{aC}O_{2}}$ when ${\dot{V}}_{CO_{2}}$ changes are small (in this case, ∼3‐fold above rest); and (b) the authors considered lack of statistically significant difference as ‘no change’, whereas a more robust conclusion could be achieved by showing no significant difference from baseline whilst also demonstrating significant differences from predicted increases in $P_{\mathrm{aC}O_{2}}$ based upon simple mathematical models assuming only conventional CO_2_ chemofeedback (Bennett & Fordyce, 1988; Bennett & Fordyce, 1993; Mitchell, 1990).

The possibility of intrathoracic pulmonary chemoreceptors capable of detecting changes in CO_2_ flow to the lungs received its greatest support in dual extracorporeal bypass experiments isolating the pulmonary from systemic circulation in dogs. Manipulating either side of the CO_2_ flow equation (Equation 5) independently (up to 8‐fold increases in pulmonary blood flow; up to 75 mmHg pulmonary arterial $P_{CO_{2}}$) increased ${\dot{V}}_{E}$ in direct proportion to CO_2_ flow whilst preserving $P_{\mathrm{aC}O_{2}}$ at baseline levels (Green & Sheldon, 1983; Sheldon & Green, 1982). These effects were abolished by bilateral cervical vagotomy.

The ‘cardiodynamic hyperpnoea’ theory was proposed from observations of an immediate increase in ${\dot{V}}_{E}$ (and maintenance of end‐tidal $P_{CO_{2}}$) in response to increased cardiac output caused by intravenous isoprenaline infusion or cardiac pacing in anaesthetised and unanaesthetised dogs (Wasserman et al., 1974). Mechanical distention of the heart and pulmonary arteries may reflexively stimulate breathing during exercise, but again, opposing evidence exists (Orr et al., 1988). Banner et al. (1988) observed blunted cardiovascular responses but normal ventilatory responses during voluntary and electrically induced leg exercise in pulmonary denervated heart–lung transplant recipients. The absence of cardiopulmonary afferents in this population casts doubt on the conclusions of Sheldon and Green, and the notion that the lungs are a site for mixed‐venous CO_2_ sensation. The ‘vascular distention’ hypothesis posits that changes in cardiac output could be detected by systemic (limb) smooth muscle mechanoreceptors, which activate thin‐fibre group III/IV afferents (Haouzi, 2014; Haouzi et al., 1999). Thus, CO_2_ flow to the lungs may be detected through means other than mixed‐venous chemoreceptors, such as changes in local muscle blood flow (and cardiac output).

Since the late 19th century, it has been known that muscle activity stimulates breathing (Johansson, 1894). Evidence for the role of muscle sensory afferent neurons in exercise hyperpnoea comes from classical papers by Comroe and Schmidt (1943), Coote et al. (1971), McCloskey and Mitchell (1972), and Kao (1963). Inspired by the work of Alam and Smirk (1937), Comroe and Schmidt (1943) observed an increase in ventilation during post‐exercise ischaemia, suggesting a metabolite‐induced reflex originating in the exercising muscles. The cross‐circulation experiments of Kao (1963) isolated the effects of muscle afferents from central neurogenic and blood‐borne breathing stimuli in anaesthetised dogs. Electrical stimulation of the hind limbs of a neurologically intact (i.e., ‘neural’) dog with hind‐limb venous blood diverted to a second, non‐exercised (i.e., ‘humoral’) dog via interposed abdominal anastomoses elicited immediate hyperventilation in the neural dog, which was abolished by spinal cord transection. Coote et al. (1971) demonstrated that electrical stimulation of ventral roots S6–L1 increased ventilation, heart rate and blood pressure concurrent with tetanic hind‐limb muscle contraction in anaesthetised cats. These responses were reduced by dorsal root transection and muscle paralysis. McCloskey and Mitchell (1972) then noted that the exercise pressor reflex persisted when large myelinated (group I/II) fibres were prevented from transmitting sensory information to the spinal cord via anodal blockade of dorsal roots. Conversely, the pressor reflex was arrested by local anaesthetic blockade of dorsal roots that preferentially supresses sensory inputs from small myelinated and unmyelinated (group III/IV) fibres. In humans, the steady‐state exercise ventilatory response is blunted by lumbar epidural fentanyl (μ‐opioid receptor agonist) injections that partially block sensory input from the exercising limbs (Amann et al., 2010; Amann et al., 2011). On balance, available evidence suggests that muscle afferents do contribute to exercise hyperpnoea.

However, once again, conflicting evidence exists. For example, Cross et al. (1982) confirmed that spinal cord transmission was not necessary for hyperpnoea during electrically induced hind‐limb activation in anaesthetised dogs. Further counter‐evidence comes from studies using percutaneous electrical stimulation of the quadriceps muscles to simulate aspects of exercise in intact (Asmussen et al., 1943) and paraplegic humans (Adams et al., 1984; Asmussen et al., 1943; Brice et al., 1988; Brown et al., 1990). In each instance, subjects displayed a ‘normal’ exercise hyperpnoea. However, muscle stimulation does not stimulate all, or even the most important, aspects of spontaneous exercise since it eliminates the possibility of a feedforward central neurogenic drive to breathe. What is more, the magnitude of increase in ${\dot{V}}_{CO_{2}}$ during muscle stimulation is limited, making it difficult to discriminate between isocapnic‐ versus chemoreflex‐driven hyperpnoea (Bennett & Fordyce, 1988).

In summary, although there is evidence for both central and peripheral neurogenic contributions to exercise hyperpnoea, none of the evidence to date is without controversy and none of the claimed mechanisms (even in combination with $P_{\mathrm{aC}O_{2}}$ feedback) are sufficient to explain exercise hyperpnoea. New hypotheses and/or layers of regulation must be considered.

### 3.3 Adaptive control

The combination of (as yet unknown) feedforward and (CO_2_) feedback neural mechanisms can explain exercise hyperpnoea in all vertebrate species studied to date, at least in normal conditions. However, physiological conditions inevitably change throughout life, requiring adaptation of the neural system controlling breathing to assure regulation of $P_{\mathrm{aC}O_{2}}$ with the same precision from rest to exercise (Mitchell, 1990; Mitchell et al., 1984). With growth and development, gain or loss of body mass, pregnancy, the onset of disease or injury, and the transition to old age, a fixed and immutable feedforward exercise stimulus cannot produce the precise blood‐gas regulation observed, even after fine‐tuning via chemofeedback. When conditions change, adaptive control makes short‐ and long‐term adjustments in properties of feedforward and/or feedback mechanisms to restore homeostasis (Priban & Fincham, 1965). Two distinct but related mechanisms of adaptive control in the exercise ventilatory response are modulation and plasticity (Babb et al., 2010; Mitchell & Babb, 2006). It has been proposed that exercise hyperpnoea may even represent a learned response guided by accumulating life experience in development, consistent with Somjen's memory theory proposed to account for missing error signals (Somjen, 1992).

Several lines of evidence support lifetime adjustments to the exercise ventilatory response. First, lung mechanics and gas exchange characteristics degrade with age, increasing physiological dead space and *V*_D_/*V*_T_. Despite this challenge, elderly individuals increase the slope of the relationship between ${\dot{V}}_{E}$ and ${\dot{V}}_{CO_{2}}$ as required (Equation 2) to regulate $P_{\mathrm{aC}O_{2}}$ from rest to exercise with similar precision to young adults (Johnson et al., 1994). Second, although adults with congestive heart failure also exhibit an increased *V*_D_/*V*_T_ due to widening ventilation–perfusion inequality, they too augment their exercise ventilatory responses to preserve $P_{\mathrm{aC}O_{2}}$ regulation (Sullivan et al., 1988). Third, in carotid denervated ponies, hyperventilation during work transitions is less 1–2 years post‐surgery versus 2–4 weeks post‐surgery (Pan et al., 1998). Mechanisms of these adjustments have never been formally addressed, although similar regulation occurs in other conditions that increase resting ventilatory drive, such as serotonin depletion (Mitchell et al., 1984), peripheral chemoreceptor stimulation via domperidone (Schaefer & Mitchell, 1989) or increased respiratory dead space (Mitchell, 1990). The uniformity of these adjustments points to an adaptive neural control system capable of modulation and/or plasticity (Mitchell et al., 1990; Priban & Fincham, 1965)

Influenced by engineering principles of systems control theory (Francis & Wonham, 1975), the internal model paradigm described by Poon et al. (2007) posits that the respiratory controller continuously integrates sensorimotor (afferent and efferent) signals during exercise and adapts according to an ‘optimality principle’. Whilst the ‘self‐tuning’ hypothesis is difficult to test experimentally, it does allude to modulation and plasticity in the neural system controlling breathing. Demonstrated examples of adaptive control in exercise hyperpnoea includes ‘short‐term modulation’ (STM) and ‘long‐term modulation’ (LTM) of the exercise ventilatory response (Bach et al., 1993; Martin & Mitchell, 1993; Mitchell, 1990; Mitchell et al., 1984; Mitchell et al., 1990; Wood et al., 2003; Wood et al., 2008).

#### 3.3.1 Defining neuromodulation and neuroplasticity in the exercise ventilatory response

**FIGURE 4:**
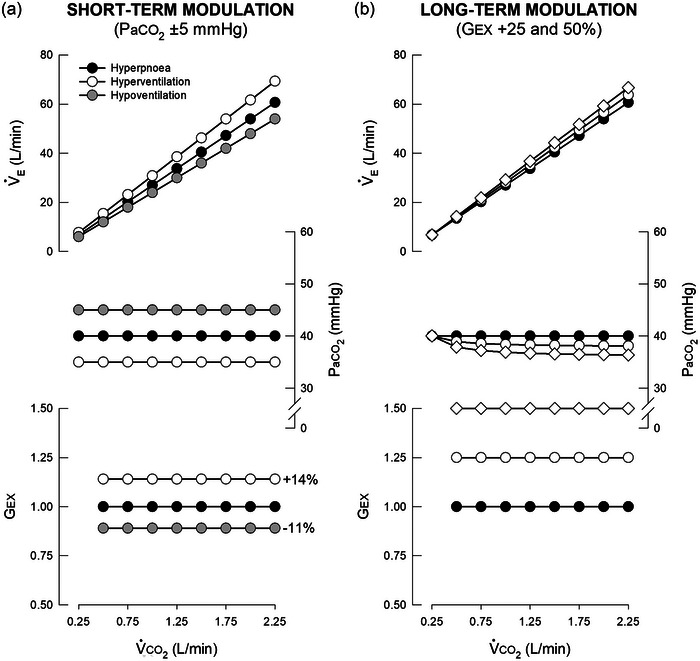
Short‐ and long‐term modulation of the exercise ventilatory response. Black symbols represent an isocapnic hyperpnoea, white symbols represent hyperventilation, and grey symbols represent hypoventilation at rest. (a) Required (active) changes in the feedforward exercise gain (*G*_EX_) and the resulting changes in system gain (${\dot{V}}_{E}$:${\dot{V}}_{CO_{2}}$ slope) to regulate arterial CO_2_ pressure ($P_{\mathrm{aC}O_{2}}$) with the same precision from rest to exercise at different $P_{\mathrm{aC}O_{2}}$ set‐points. To regulate $P_{\mathrm{aC}O_{2}}$ at 45 mmHg, *G*_EX_ must decrease by 11% from the value required to regulate $P_{\mathrm{aC}O_{2}}$ at 40 mmHg. To regulate $P_{\mathrm{aC}O_{2}}$ at 35 mmHg, *G*_EX_ must increase by 14%. Changes in the $P_{\mathrm{aC}O_{2}}$ set‐point and immediate (appropriate) changes in *G*_EX_ to enable similar $P_{\mathrm{aC}O_{2}}$ regulation demonstrate short‐term modulation. (b) A 25% and 50% increase in *G*_EX_ with corresponding changes in the ${\dot{V}}_{E}$:${\dot{V}}_{CO_{2}}$ slope of +5% and +10%, respectively. The former is identical to Figure 1c, whereby CO_2_ chemofeedback layers onto the primary feedforward exercise stimulus to produce only a ∼2 mmHg decrease in $P_{\mathrm{aC}O_{2}}$ from rest at a ${\dot{V}}_{CO_{2}}$ of 2.25 L/min. The latter represents an additional layer of neuroplasticity (i.e., long‐term modulation), denoted by diamonds, causing a ∼4 mmHg decrease in $P_{\mathrm{aC}O_{2}}$ from rest. ${\dot{V}}_{CO_{2}}$, rate of CO_2_ production; ${\dot{V}}_{E}$, minute ventilation.

Inclusive definitions of modulation and plasticity in respiratory control can be found elsewhere (Babb et al., 2010; Mitchell & Babb, 2006; Mitchell & Johnson, 2003). Briefly, modulation is a change in the control system that occurs when the initiating stimulus is present, but reverses rapidly once that stimulus is removed (minutes). Plasticity, on the other hand, is a change that outlasts the initiating stimulus, persisting hours, days or even a lifetime after the stimulus has ended. With these simple definitions in mind, STM is an immediate change in the exercise ventilatory response that reverses when the inducing stimulus is removed. In contrast, LTM is a persistent change in the exercise ventilatory response beyond the duration of the inducing stimulus. Thus, the term ‘LTM’ as originally defined is somewhat misleading since it is an example of neuroplasticity. Confusion in definitions arose because the field was not welcoming to the concept of plasticity at the time LTM was first described (Martin & Mitchell, 1993), and definitions of modulation and plasticity in respiratory control had not yet been formalised (Mitchell & Johnson, 2003). The effects of STM and LTM on ${\dot{V}}_{E}$:${\dot{V}}_{CO_{2}}$ coupling during exercise are illustrated conceptually in Figure 4.

#### 3.3.2 Short‐term modulation of the exercise ventilatory response

Short‐term modulation can be demonstrated in goats and humans with acute presentation of external dead space (Mitchell, 1990; Wood et al., 2008). Increased dead space using a wide bore tube with hyperoxic background conditions represents a CO_2_ load with unique properties in comparison with increasing inspired CO_2_ concentrations (Mitchell & Osborne, 1980). In goats, increased dead space (200–600 ml) increases *V*_D_/*V*_T_, reduces ${\dot{V}}_{A}$ and increases resting $P_{\mathrm{aC}O_{2}}$ (∼1 to 4 mmHg). With mild or moderate treadmill exercise, $P_{\mathrm{aC}O_{2}}$ is regulated slightly below (∼2 mmHg) this new baseline level through increased system gain (Mitchell, 1990). Increased system gain was partitioned into the feedforward exercise (*G*_EX_, Equation 6) versus feedback gain (*S* = Δ${\dot{V}}_{E}$/Δ$P_{\mathrm{aC}O_{2}}$) in accordance with a simple mathematical model (Mitchell, 1990):

(6) $$G_{\mathrm{EX}}=\left(\Delta {\dot{V}}_{E}/\Delta {\dot{V}}_{CO_{2}}\right)-S\times \left(\Delta P_{\mathrm{aC}O_{2}}/\Delta {\dot{V}}_{CO_{2}}\right)$$

In this model, increased system gain (*G*_SYS_ = Δ${\dot{V}}_{E}$/Δ${\dot{V}}_{CO_{2}}$) with increased dead space could be accounted for by increased *G*_EX_ with unchanging *S*. Since *G*_SYS_ increased within a single exercise trial and was reversed in subsequent trials exercise without dead space, *G*_EX_ was increased by STM.

In healthy young men, a similar increase in *G*_SYS_ occurs during mild exercise with added dead space, regulating end‐tidal $P_{CO_{2}}$ with the same precision from rest to exercise at its new elevated resting level (Wood et al., 2008, 2009). In subsequent studies, STM was reported in elderly men (Wood et al., 2010), younger and elderly women (Wood et al., 2011), and people with obesity and obstructive sleep apnoea (Bernhardt et al., 2017). Thus, external dead space (and increased *V*_D_/*V*_T_) elicits STM, possibly accounting for the increased exercise ventilatory responses characteristic of old age and heart failure (see above).

After thoracic dorsal rhizotomy from T2–T12, goats are no longer able to compensate for increased respiratory dead space during exercise with STM, at least initially (Mitchell et al., 1990). With a respiratory mask in place, treadmill‐trained goats exhibit overt ventilatory failure and hypercapnia, although the magnitude of failure diminished with each successive exercise trial. Strikingly, goats were once again able to generate a normal exercise hyperpnoea after multiple exercise trials. Thus, although these data may suggest some inability to elicit STM, another mechanism intervenes and restores function. Gradual restoration of exercise hyperpnoea with repeated exercise trials inspired studies that led to the discovery of LTM (Martin & Mitchell, 1993). In *post mortem* studies on the goats with thoracic dorsal rhizotomy, it was verified that regeneration of thoracic sensory afferent neurons could not be observed. Nevertheless, elevations in cervical and thoracic monoamine concentrations (e.g., serotonin, noradrenaline and dopamine) (Mitchell et al., 2000) led to further exploration of mechanisms giving rise to functional recovery. In rats, cervical dorsal rhizotomy increases serotonin (Kinkead et al., 1998) and neurotrophic factors (Johnson et al., 2000), and enhances phrenic motor plasticity following acute intermittent hypoxia (Kinkead et al., 1998). These results are consistent with the idea that monoamines (e.g., serotonin) play key roles in respiratory motor plasticity and contribute to functional recovery of exercise hyperpnoea after thoracic sensory denervation.

In concordance, systemic administration of a broad‐spectrum serotonin receptor antagonist (methysergide) abolishes STM with increased dead space in goats (Bach et al., 1993). Since methysergide and a more selective serotonin 5‐HT_2A_ receptor antagonist, ketanserin, delivered to the thoracic cerebrospinal fluid via an indwelling catheter abolish STM (Mitchell et al., 2008), spinal serotonin receptor activation is necessary for STM.

#### 3.3.3 Long‐term modulation of the exercise ventilatory response

Long‐term modulation occurs in goats and humans in response to repeated trials of combined exercise with CO_2_ loading (dead space or inspired CO_2_). After repeated trials of hypercapnic exercise, LTM manifests as profound hyperventilation during initial exercise trials without CO_2_ loading (Martin & Mitchell, 1993; Wood et al., 2003). However, with subsequent (unloaded) trials, the exercise ventilatory response returns to normal, demonstrating a lasting but reversible form of learning or memory in the exercise ventilatory response.

**FIGURE 5:**
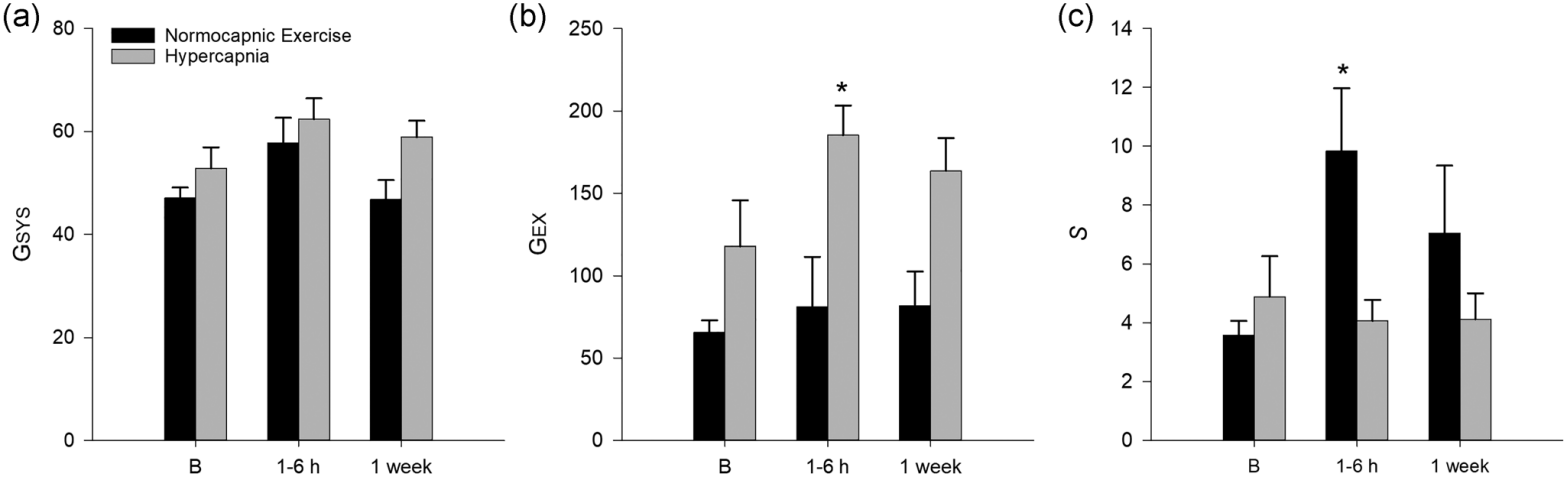
Long‐term modulation of the exercise ventilatory response in goats. Changes in system gain (*G*_SYS_ = Δ${\dot{V}}_{E}$/Δ${\dot{V}}_{CO_{2}}$; a), feedforward gain (*G*_EX_ = [(Δ${\dot{V}}_{E}$/Δ${\dot{V}}_{CO_{2}}$) − *S*(Δ$P_{\mathrm{aC}O_{2}}$/Δ${\dot{V}}_{CO_{2}}$)]; b), and feedback gain (*S* = Δ${\dot{V}}_{E}$/Δ$P_{\mathrm{aC}O_{2}}$; c) are shown during moderate exercise (4 km/h, 5% incline) in goats after 14–16 trials of normocapnic exercise training (black bars) and 14–16 trials of hypercapnic training (grey bars). Time points are baseline (B), 1–6 h post‐training and 1 week post‐training. These data demonstrate that *G*_EX_ is increased to some extent after hypercapnic training at rest, but not after repeated exercise without hypercapnia (b). Reproduced from data published only in abstract form (Martin & Mitchell, 1992; Martin et al., 1993).

Although LTM is induced by repeated exercise paired with CO_2_ loading, it is not observed following repeated bouts of normocapnic (Martin & Mitchell, 1992) or hypoxic exercise (Turner et al., 1995). On the other hand, repeated CO_2_ loading at rest (without exercise) partially elicits LTM (Martin et al., 1993), as shown in Figure 5. The capacity for LTM after repeated hypercapnia at rest, and the failure of repeated hypoxic exercise to elicit LTM, are perplexing since they suggest that the explicit pairing of hypercapnia with exercise, and carotid body chemoreceptor feedback, are not necessary for full LTM expression.

Attempts to replicate LTM in humans are somewhat inconsistent. Whilst some studies report LTM in phase I (Helbling et al., 1997; Turner & Stewart, 2004; Turner & Sumners, 2002) and/or phases II or III (Reed & Coates, 2001; Wood et al., 2003), others do not (Al‐Harbi et al., 2001; Cathcart et al., 2005; Cathcart et al., 2010; Moosavi et al., 2002; Sumners & Turner, 2003). Many factors could explain the discrepancy in findings, particularly the number and duration of training trials, severity of hypercapnia, mode of inducing hypercapnia (e.g., dead space vs. inspired CO_2_), participant training status, and history of physical activity. Notably, no human investigation to date directly measured $P_{\mathrm{aC}O_{2}}$ or respiratory mechanics, and most fail to report the slope of the ${\dot{V}}_{E}$:${\dot{V}}_{CO_{2}}$ relationship (i.e., *G*_SYS_) or *G*_EX_. Thus, conditions necessary for LTM in humans are unclear. Other open questions are the need for explicit association of hypercapnia and exercise (Turner & Sumners, 2002), and the task specificity of exercise training (Sumners & Turner, 2003). In the most robust protocol to date (Wood et al., 2003), demonstration of LTM was compelling since it presented the highest number of hypercapnic exercise trials (*n *= 70) among published studies. An identical protocol was used to demonstrate learning in the exercise cardiovascular response. Repeated associations of moderate exercise with neck suction to activate arterial baroreceptors attenuated increases in systolic blood pressure during subsequent exercise without neck suction for up to 2 days post‐training (Herigstad et al., 2007).

As with STM, serotonin is believed to play a key role in LTM. In goats with serotonin depletion due to inhibition of the rate limiting enzyme in serotonin biosynthesis, tryptophan hydroxylase, LTM is no longer observed (Johnson & Mitchell, 2001). Hypercapnia (particularly when combined with exercise) activates brainstem raphe serotonergic neurons, releasing serotonin in the spinal cord. In a single exercise trial, serotonin release triggers STM. With repeated spinal serotonin release, LTM is expected. By way of analogy in anaesthetised rats, repeated cervical spinal serotonin receptor activation elicits long‐term facilitation of phrenic motor neuron activity (MacFarlane & Mitchell, 2009; MacFarlane et al., 2011). Thus, STM may transition to LTM after repeated trials (and serotonin receptor activation).

In our working model, spinal serotonin release and 5‐HT_2_ receptor binding on respiratory motor neurons activates intracellular kinases that increase motor neuron excitability by closing potassium channels (i.e., STM) and/or phosphorylation of glutamate receptors that strengthen synaptic inputs onto respiratory motor neurons (i.e., LTM). Increased excitability potentiates motor neuron output for the same descending respiratory drive, augmenting ${\dot{V}}_{E}$ at rest and during exercise. Reports that serotonin depletion by tryptophan hydroxylase inhibition with *p*‐chlorophenylalanine abolishes both STM (Bach et al., 1993) and LTM (Johnson & Mitchell, 2001) suggest a common mechanism. How repeated serotonin receptor activation engages this mechanism is unknown, but may be akin to the transition from phrenic long‐term facilitation (i.e., plasticity) to enhanced phrenic long‐term facilitation (i.e., metaplasticity) when rats are exposed to acute intermittent hypoxia on consecutive days (Fields & Mitchell, 2015).

In summary, the exercise ventilatory response exhibits considerable modulation and plasticity—features infrequently considered in earlier discussions of exercise hyperpnoea. Below, we address the exercise hyperpnoea dilemma, reasoning that the primary feedforward exercise stimulus is under constant adaptive control, and that mechanisms of learning, memory and plasticity may explain the near‐perfect $P_{\mathrm{aC}O_{2}}$ regulation during moderate exercise.

## 4 PART III: THE EXERCISE HYPERPNOEA DILEMMA

Major challenges must be met before we can solve the exercise hyperpnoea dilemma, including: (a) identifying factors that drive exercise hyperpnoea and accurately ‘calibrate’ ${\dot{V}}_{A}$ across a wide ${\dot{V}}_{CO_{2}}$ range; (b) generating unequivocal evidence that these factors are both necessary and sufficient for a normal exercise hyperpnoea in intact animal models or humans; and (c) differentiating between the primary exercise stimulus versus compensatory mechanisms that substitute when the primary stimulus is impaired (referred to as ‘redundancy’).

Notwithstanding the challenges listed above and the long history of attempts to solve the exercise hyperpnoea dilemma, there is reason for optimism since exceptional strides have been made in the field of neuroscience over the past few decades and concepts of modulation and plasticity are now established in the neural control of breathing (Feldman et al., 2003; Mitchell & Baker, 2022; Vose et al., 2022). Merging new knowledge with technical neuroscience innovations creates exciting new opportunities to probe specific neural substrates, cells and circuits that drive (or at least contribute to) exercise hyperpnoea.

### 4.1 A new perspective on exercise hyperpnoea

A principal aim of this review is to reframe our approach to understanding exercise hyperpnoea. We propose that adaptive control (i.e., plasticity/learning) adjusts exercise hyperpnoea so that it remains appropriate in the face of ever‐changing physiological conditions from birth to the end of life. The goal of an adaptive exercise hyperpnoea is to regulate $P_{\mathrm{aC}O_{2}}$ with the same precision from rest to exercise despite unavoidable changes in intrinsic respiratory system characteristics, such as lung compliance or pulmonary gas exchange that accompanies development and ageing. Other disturbances that challenge the accuracy of exercise hyperpnoea include permanent or reversible changes in body weight, diet, pregnancy and changes in ambient conditions (e.g., high altitude).

As a corollary, we posit that modulation and plasticity compensate for the onset of lung (e.g., asthma) or neural injury/diseases (e.g., spinal cord injury, ALS) that impair breathing function. Hence, increased understanding of how respiratory modulation and/or plasticity influences the exercise ventilatory response across the lifespan is of paramount significance from a translational perspective, particularly as it pertains to the development of novel therapeutics to preserve/restore breathing in clinical disorders that cause exercise intolerance due to respiratory compromise. Although speculative, the hypotheses presented herein are put forth in the hope they will stimulate original investigations into the nature and mechanisms of exercise hyperpnoea—the largest (yet still unexplained) ventilatory response experienced by all individuals during normal life.

### 4.2 Learned respiratory behaviours

Exercise hyperpnoea, like other homeostatic regulatory behaviours, may gradually accumulate throughout development in response to repeated error signals (Somjen, 1992). In the newborn, CO_2_ chemoreception is already intact, although it can take a week or more to come ‘online’ in some species (Davis et al., 2006; Stunden et al., 2001; Wickstom et al., 1999). Thus, repeated CO_2_ perturbations associated with physical activity may trigger neuroplasticity, adjusting system responses to meet changing needs. Once established, neuroplasticity can once again adjust the exercise ventilatory response if new error signals indicate the need for further system refinement.

If physical activity is associated with repeated error signals near birth, it may evoke neuroplasticity (i.e., learning) and guide the development of (or refine) exercise hyperpnoea. However, bilateral carotid denervation in neonatal goats (1–3 days of age) does not impact the adult (3–18 months of age) exercise ventilatory response (Lowry et al., 1999), casting some doubt on the developmental hypothesis. Nevertheless, the reliance of LTM on CO_2_ chemoreception (Mitchell et al., 2008; Turner & Mitchell, 1997; Turner et al., 1995) versus other forms of respiratory motor plasticity that rely on O_2_ chemoreception (Feldman et al., 2003; Mitchell & Baker, 2022) suggests that this question has not been adequately tested.

A prominent model to study learned respiratory behaviours is the gill withdrawal reflex of the marine invertebrate *Aplysia californica*, whereby many forms of learning have been identified, such as sensitization, habituation and classical conditioning (Walters et al., 1979, 1982). With classical conditioning, learning results from the explicit association of a conditioned stimulus with the unconditioned stimulus (Pavlov, 1927). In *Aplysia*, the conditioned stimulus (siphon touch) elicits only weak siphon and gill withdrawal when presented alone. However, when paired with an aversive (unconditioned) stimulus such as a tail shock, the response to siphon touch is sensitised. If repeated, paired associations are presented (∼3 to 15 trials), long‐lasting memory is formed (Carew et al., 1981; Carew et al., 1983). Similar to respiratory motor plasticity in vertebrates (e.g., phrenic long‐term facilitation and LTM), serotonin mediates the classically conditioned gill withdrawal reflex in *Aplysia* (Glanzman et al., 1989).

In unanaesthetised cats, Orem and Netick (1986) paired an auditory tone (conditioned stimulus) with a noxious respiratory stimulus (ammonia inhalation; unconditioned stimulus), which terminates inspiration and prolongs expiration. When the conditioned and unconditioned stimuli were repeatedly paired, the conditioned tone triggers apnoea, demonstrating a form of associative learning and memory in the neural circuits controlling breathing. In humans, Gallego and Perruchet (1991) showed that eight pairings of an auditory tone (conditioned stimulus) with hypoxia (unconditioned stimulus) for 12 breath cycles elicits a conditioned hyperventilation. Based upon this evidence for classical, associative conditioning of respiratory motor behaviours, it is worthwhile to contemplate what is known about associative conditioning mechanisms in non‐respiratory motor behaviours, such as the conditioned eye‐blink response in rabbits. In this model of associative motor conditioning, the cerebellum has been implicated as a key site of memory engram storage (Krupa et al., 1993; McCormick et al., 1981).

### 4.3 Could a cerebellar memory engram encode the hyperpnoea of exercise?

In their comprehensive review of respiratory control during exercise, Forster and colleagues stated, ‘To our knowledge it has not been postulated nor is there rationale or evidence to suggest that cerebellar nuclei are the primary source of the stimulus for the exercise hyperpnoea’ (Forster et al., 2012). On the other hand, there is also no evidence to rule out a salient cerebellar role since research on this topic has been minimal. Here, we outline evidence that the cerebellum is more than a site of sensorimotor integration and coordination, as it has well‐documented roles in multiple forms of motor learning. Exercise hyperpnoea is a complex motor behaviour that requires contributions from many well‐coordinated respiratory muscles. As a highly orchestrated motor behaviour modified by neuroplasticity, there is sufficient evidence to consider that the memory engram for exercise hyperpnoea at least partially resides within the cerebellum. For the purposes of this review, an engram is defined as physical and/or chemical changes elicited by learning that underlie newly formed memory associations (Josselyn & Tonegawa, 2020).

#### 4.3.1 Role of the cerebellum in motor learning

The cerebellum contains approximately 69 billion neurons—over 80% of all neurons in the brain (Azevedo et al., 2009). The cerebellum receives inputs carrying information about outgoing motor commands from the motor cortex and brainstem, as well as sensory information via the brainstem and spinal cord (Brodal, 1981). These anatomical connections exhibit functional topography (Stoodley & Schmahmann, 2018). Thus, the cerebellum is an ideal network for sensorimotor learning (Brindley, 1964; Hansel et al., 2001) and functions as an ‘array of adjustable motor pattern generators’ that stores and retrieves learned motor behaviours (Houk, 1987).

Evidence that the cerebellum is critical for normal motor function and the formation of motor memories is shown by individuals with cerebellar degeneration that have persistent deficits in visuomotor learning (Martin et al., 1996), speech adaptation (Parrell et al., 2017) and locomotor adaptation (Morton & Bastian, 2006). These deficits arise from impaired sensorimotor memory formation (Hadjiosif et al., 2023). Although important, such observations do not shed light on mechanisms of cerebellum‐dependent motor learning.

Guided by detailed study of cerebellar neural networks by Eccles et al. (1967), Marr (1969) and Albus (1971) recognised that cerebellar synaptic connections are modified by experience. The crux of the Marr–Albus model of associative cerebellar memory is that parallel fibre‐to‐Purkinje cell synapses undergo long‐lasting changes due to concurrent climbing fibre activation, which serves as the ‘teacher’ for new motor behaviours through error signal detection. An excellent example of cerebellar associative memory is the conditioned eye‐blink response in rabbits (Houk et al., 1996; Krupa et al., 1993; McCormick et al., 1981).

Conditioned eye‐blink responses occur when a conditioned stimulus (e.g., audio tone) is repeatedly paired with an aversive, unconditioned stimulus (e.g., air‐puff into the eye). After explicitly paired presentations with a specific time interval between the conditioned and unconditioned stimuli, the conditioned stimulus subsequently evokes the conditioned response (i.e., eye‐blink). Cells that comprise the engram for this simple motor memory are localised in the cerebellar interpositus nucleus—a site that is both necessary and sufficient for classically conditioned eye‐blink responses (Christian & Thompson, 2003).

Recent experiments established that cerebellar Purkinje cells encode signals associated with the predicted sensory consequences of movement versus actual movement (Pasalar et al., 2006; Popa et al., 2012). Sensory prediction errors occur if there is a mismatch between the predicted sensory consequence of movement computed by the feedforward controller and actual sensory feedback. Brooks et al. (2015) recorded neural responses of the rostral fastigial nucleus in two adult rhesus monkeys during voluntary head movements with unexpected perturbations (resistive torque). The sensitivities (i.e., response gain) of rostral fastigial nucleus neurons dynamically tracked the initial introduction of error signals and its subsequent decline during motor learning, mirroring responses of the vestibular nuclei. These results elegantly demonstrate that deep cerebellar nuclei learn to expect the unexpected, ensuring that movements remain accurately calibrated over time.

#### 4.3.2 Role of the cerebellum in respiratory control

The cerebellum has elaborate direct and indirect projections to regions relevant to respiratory motor control (Krohn et al., 2023), including the nucleus of the solitary tract (Andrezik et al., 1984), rostral ventral respiratory group (Gaytan & Pasaro, 1998), trigeminal motor nucleus (Judd et al., 2021), facial nucleus (Moolenaar & Rucker, 1976), hypoglossal motor nucleus (Guo et al., 2020), Kölliker–Fuse nucleus (Fujita et al., 2020), locus coeruleus (Teune et al., 2000), dorsal raphe nucleus (Cavdar et al., 2018), periaqueductal grey (Anand et al., 1959; Frontera et al., 2020; Koutsikou et al., 2014) and hypothalamus (Dietrichs et al., 1994). Although the cerebellum's role in respiratory control is under‐studied, it is known that deep cerebellar nuclei modulate breathing (Xu & Frazier, 1997, 2002), and are involved in certain forms of respiratory motor plasticity. For example, phrenic long‐term facilitation induced by acute intermittent hypoxia is obliterated by removing the cerebellar vermis (i.e., the part of the spinocerebellum containing the rostral fastigial nucleus) (Hayashi et al., 1993). As phrenic long‐term facilitation is cerebellar and serotonin‐dependent (Feldman et al., 2003; Mitchell et al., 2001), it may share common features with serotonin‐dependent LTM (Mitchell & Babb, 2006; Mitchell et al., 2008).

Electrical stimulation of the rostral fastigial nucleus in anaesthetised cats increases respiratory frequency and/or amplitude (Bassal & Bianchi, 1982). Similarly, tachypnoea is observed during electrical stimulation within close proximity of the rostral fastigial nucleus in humans (Hirano et al., 1993). The rostral fastigial nucleus receives and integrates information from respiratory afferent neurons, including chemoreceptors (Lutherer et al., 1989). Additional evidence suggests that the rostral fastigial nucleus is CO_2_ sensitive, as demonstrated by increased respiratory amplitude after localised acetazolamide microinjections in rats (Xu et al., 2001).

There are data to suggest that the rostral fastigial nucleus may also play a role in the neural control of breathing during exercise. In goats, bilateral neurotoxic lesions of the rostral and caudal fastigial nucleus attenuates the normal exercise‐induced hypocapnia by 1.3–2.8 mmHg during mild steady‐state treadmill exercise (Martino et al., 2006). In these experiments, only ∼55% of fastigial neurons were destroyed, and post‐lesion studies were delayed by more than 1 week when the goats had recovered walking ability. Two important considerations when interpreting these findings are: (a) elimination of exercise‐induced hypocapnia (∼2 mmHg) represents a nearly 25% reduction in the exercise feedforward stimulus once the inhibitory role of chemofeedback is accounted for (Mitchell, 1990); and (b) since ∼50% of fastigial neurons were lesioned, spared neurons may have compensated, similar to respiratory motor neuron compensation in ALS (Seven & Mitchell, 2019). Thus, cerebellar contributions to exercise hyperpnoea may have been underestimated. In humans undergoing functional imaging, the cerebellum (and possibly the fastigial nucleus) increases activity during active inspiration (Colebatch et al., 1991), active expiration (Ramsay et al., 1993), imagined exercise (Thornton et al., 2001), voluntary hyperpnoea (McKay et al., 2003) and asphyxia (McKay et al., 2010).

Collectively, these studies give credence to the possibility of a major cerebellar role in exercise hyperpnoea, particularly any learned component. The cerebellum is important for somatic motor learning (as envisioned by Marr and Albus) and discrete areas of the cerebellum (e.g., fastigial nucleus) are clearly engaged in the control of breathing. However, the specific role of the cerebellum in acquisition or storage of a learned exercise hyperpnoea has never been formally tested to our knowledge.

### 4.4 Working hypothesis

Here, we pose the hypothesis that cerebellar learning is obligatory for normal development and maintenance of exercise hyperpnoea in humans and other mammals. Our hypothesis is largely based on demonstrations that exercise hyperpnoea exhibits plasticity in adult mammals (Martin & Mitchell, 1993; Mitchell et al., 1990), that the cerebellum contributes to respiratory control, including forms of respiratory ‘memory’ (Hayashi et al., 1993), and that deep cerebellar nuclei are crucial in other forms of motor learning (Clark et al., 1992; Krupa & Thompson, 1995, 1997; Krupa et al., 1993). For example, the cerebellar interpositus nucleus is central to both the acquisition and retention of conditioned eye‐blink responses (Krupa et al., 1993). Once acquired, the conditioned response is eliminated and then restored by reversibly inhibiting nucleus interpositus neurons. In contrast, inactivation of relevant brainstem motor nuclei also prevents the unconditioned reflex, without impairing acquisition of the conditioned response (Krupa et al., 1996). Thus, strong evidence supports the stance that the engram (vs. unconditioned reflex) is stored in the cerebellar interpositus nucleus. Similar approaches using more contemporary techniques to activate/inactivate target neurons without impacting off‐target neurons can be used to investigate cerebellar contributions to exercise hyperpnoea.

Plasticity in the exercise ventilatory response has been shown in previous studies using repeated associations of external dead space or elevated inspired CO_2_ to elicit hypercapnia (unconditioned stimulus) with moderate exercise (conditioned stimulus) (Martin & Mitchell, 1993; Wood et al., 2003). This model of associative learning in exercise hyperpnoea may be useful to test suspected cerebellar engram locations. Powerful chemogenetic and optogenetic techniques are now available to manipulate specific neural pathways of interest and some have already begun to apply such methods to the control of breathing during exercise (Herent et al., 2023; Korsak et al., 2018). Thus, it is possible to test if a given site is sufficient (e.g., excitatory designer receptors activated by designer drugs, DREADDs) and necessary (e.g., inhibitory DREADDs) to account for exercise hyperpnoea. For instance, inactivation of the inferior olivary nucleus, climbing fibres, Purkinje cells or the rostral fastigial nucleus during versus after associative training may prevent (or minimise) the feedforward exercise stimulus, leaving residual chemofeedback‐driven ventilation characterised by elevated $P_{\mathrm{aC}O_{2}}$ from rest to exercise. Alternatively (or in addition), inhibition of these neurons may prevent the ability to acquire LTM. If inactivation of cerebellar circuits prevents LTM, activation of those same circuits should (a) increase ${\dot{V}}_{E}$ substantially at rest, driving severe hypocapnia, and/or (b) elicit LTM if paired with exercise without CO_2_ loading. Long‐term modulation would be expressed as a reversibly enhanced feedforward exercise gain and exacerbated exercise hypocapnia.

**FIGURE 6:**
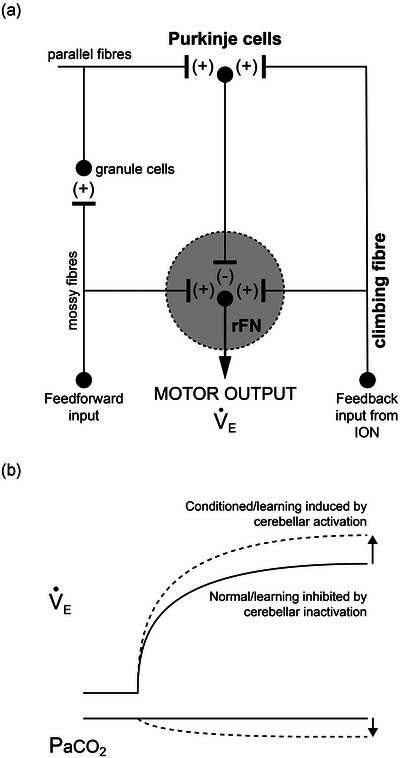
Hypothesis. (a) A diagram of a cerebellar neural network including a possible site of plasticity at the climbing fibre‐to‐Purkinje cell synapse. (b) A normal ventilatory response to mild or moderate steady‐state exercise with good regulation of arterial $P_{CO_{2}}$ ($P_{\mathrm{aC}O_{2}}$; unbroken lines). Repeated trials of hypercapnic training elicit hyperventilation during subsequent exercise trials without CO_2_ loading due to associative learning (i.e., long‐term modulation of the exercise ventilatory response; dashed lines). We hypothesise that learning can be inhibited by chemogenetic inactivation of Purkinje cells or the rostral fastigial nucleus (rFN) during conditioning or induced by optogenetic activation of the inferior olivary nucleus (ION) or the climbing fibre during exercise without need for CO_2_ loading. See text for further details. ${\dot{V}}_{E}$, minute ventilation.

Figure 6 is a simplified diagram of cerebellar neural networks depicting our working model. We propose that feedforward inputs activate mossy fibres whereas changes in $P_{\mathrm{aC}O_{2}}$ activate climbing fibres. During hypercapnic exercise, mossy and climbing fibres are activated in close temporal association. Deep cerebellar nuclei subsequently modulate breathing during exercise. Inhibitory chemogenetics targeting Purkinje cells and/or neurons in the rostral fastigial nucleus as the site of plasticity would theoretically prevent learning (i.e., acquisition of LTM), or even block the exercise hyperpnoea if it represents a learned response from birth with the engram stored at this site. Optogenetics may also be useful to target inferior olive/climbing fibre activation (simulating error signals) to test their requirement for plasticity.

### 4.5 Limitations and other considerations

Our hypotheses are highly speculative, but are presented in an effort to stimulate new research on a topic that has seemingly ‘run out of good ideas’. We do not yet know: (a) if exercise hyperpnoea is an example of life‐long learning versus a response modified by neuroplasticity; (b) if the cerebellum is implicated in LTM of the exercise ventilatory response; (c) where the engram for exercise hyperpnoea is stored and what cells types are involved; and (d) if the engram is an ensemble of widely distributed cells in multiple regions of the central nervous system, as with other forms of learning and memory, such as fear conditioning (Josselyn & Tonegawa, 2020).

## 5 SUMMARY

Our understanding of the neural control of breathing continues to expand. Recent conceptual and technological breakthroughs may allow researchers, for the first time, to precisely target specific neural pathways associated with learning, memory and plasticity in automatic (respiratory) or autonomic (cardiovascular) responses to exercise. Explaining the mechanistic basis of exercise hyperpnoea remains a great challenge facing respiratory and exercise physiologists. Julius Comroe once stated ‘… respiratory physiologists are not necessarily good neurobiologists or good control system engineers, and maybe they must be to solve the problem’ (Comroe, 1965). Nearly 60 years later, we echo this sentiment, and hope to reopen discussions that generate new ideas and renewed study of exercise hyperpnoea in the 21st century.

Our perspective is that the hyperpnoea of exercise is (continuously) adjusted throughout life, and may drive development of a normal exercise ventilatory response, or adapt a congenital, rudimentary feedforward exercise stimulus so that it generates appropriate exercise hyperpnoea despite changing conditions. Relevant conditions that require adaptation include healthy ageing, weight gain/loss, injury, onset of lung disease, sojourn to high altitude and many others. Each of these experiences requires active adjustment (i.e., modulation and/or plasticity) of the neural system driving breathing, or the superb regulation of $P_{\mathrm{aC}O_{2}}$ typical of mild or moderate exercise will degrade, limiting human performance or even life. These ideas are not adequately tested and considerable research efforts are needed to determine if they have merit.

We speculate that the memory engram encoding the hyperpnoea of exercise resides within the cerebellum. Although there are certainly other possibilities, there is ample evidence from other automatic motor systems to strongly consider this possibility. Of great importance, these and alternate hypotheses can be tested using modern neuroscience methods (and concepts) that were unavailable to previous generations of researchers interested in the exercise hyperpnoea dilemma (Swanson, 1978).

## AUTHOR CONTRIBUTIONS

Both authors have read and approved the final version of this manuscript and agree to be accountable for all aspects of the work in ensuring that questions related to the accuracy or integrity of any part of the work are appropriately investigated and resolved. Both persons designated as authors qualify for authorship, and all those who qualify for authorship are listed.

## CONFLICT OF INTEREST

The authors declare no conflicts of interest.
